# Integration of phage and yeast display platforms: A reliable and cost effective approach for binning of peptides as displayed on-phage

**DOI:** 10.1371/journal.pone.0233961

**Published:** 2020-06-01

**Authors:** Priyanka Pandya, Robert O. Sayers, Joey P. Ting, Shaghayegh Morshedian, Carina Torres, Justine S. Cudal, Kai Zhang, Jonathan R. Fitchett, Qing Zhang, Feiyu F. Zhang, Jing Wang, Jim D. Durbin, Juan J. Carrillo, Alfonso Espada, Howard Broughton, Yuewei Qian, Sepideh Afshar

**Affiliations:** 1 Department of Protein Engineering, Eli Lilly Biotechnology Center, San Diego, California, United States of America; 2 Lilly Research Laboratories, Discovery Chemistry Research and Technologies, San Diego, California, United States of America; 3 Department of Structural Biology, Discovery Chemistry Research and Technologies, Eli Lilly and Company, Indianapolis, Indiana, United States of America; 4 Department of Quantitative Biology, Discovery Chemistry Research and Technologies, Lilly Biotechnology Center, Eli Lilly and Company, Indianapolis, Indiana, United States of America; 5 Centro de Investigación Lilly, Alcobendas, Spain; 6 Lilly Research Laboratories, Recombinant Protein Generation, Indianapolis, Indiana, United States of America; Consejo Superior de Investigaciones Cientificas, SPAIN

## Abstract

Hundreds of target specific peptides are routinely discovered by peptide display platforms. However, due to the high cost of peptide synthesis only a limited number of peptides are chemically made for further analysis. Here we describe an accurate and cost effective method to bin peptides on-phage based on binding region(s), without any requirement for peptide or protein synthesis. This approach, which integrates phage and yeast display platforms, requires display of target and its alanine variants on yeast. Flow cytometry was used to detect binding of peptides on-phage to the target on yeast. Once hits were identified, they were synthesized to confirm their binding region(s) by HDX (Hydrogen deuterium exchange) and crystallography. Moreover, we have successfully shown that this approach can be implemented as part of a panning process to deplete non-functional peptides. This technique can be applied to any target that can be successfully displayed on yeast; it narrows down the number of peptides requiring synthesis; and its utilization during selection results in enrichment of peptide population against defined binding regions on the target.

## Introduction

Peptide discovery platforms such as phage display have facilitated de novo peptide discovery of hundreds of target specific peptide-phage [1–6]. The diversity of the peptide library increases the chance of identifying hits with the desired properties. Once hits are identified, they must be characterized and since it is nearly impossible to predict which peptide will maintain its binding as a free peptide, it is a recommended practice to synthesize the majority of the binders. However, chemical synthesis and characterization of each identified peptide is a costly and time-consuming endeavor and generally only very few hits can be made synthetically outside the phage context. Therefore, peptides with the most optimal properties (function, solubility, and stability) might be overlooked, if only a small pool of peptides is characterized.

To circumvent this challenge, different attempts have been made to identify functional peptides as they are displayed on-phage. If successful, only the functional peptides need to be synthesized. One approach includes competition ELISA and MSD phage-based assays. However, we have not observed a direct correlation between functionality of the phage-displayed versus free peptides across multiple projects. Another approach is to fuse the peptides to another carrier [7]. This approach is also costly and resource intensive and while it addresses multivalency on-phage, it does not eliminate issues around context-dependent binding. A third approach includes *in vitro* translation (IVT) of peptides. Due to low peptide yield, this approach is not suitable for functional analysis of the peptides and can only confirm binding of the peptide outside the phage context. Moreover, affordable IVT systems are not suitable for translation of short peptides; hence, peptides need to be fused to other scaffolds/tags to enable translation and quantification. The fourth approach is to identify peptides that bind to the region of interest by alanine mutagenesis of the target. However the process of cloning, expressing, purifying, and characterizing each alanine mutation is also laborious and time-consuming [8]. Therefore, we decided to display the antigen on yeast and use flow cytometry to screen phage hits in the phage context. Specifically, we displayed wild-type IL-23 and its alanine variants on yeast to test the feasibility of our approach. IL-23 is a heterodimeric cytokine comprised of p19 and p40 subunits and plays a key role in several autoimmune diseases [9–13]. We first selected our libraries against wild-type recombinant IL-23 to enrich a population of target specific peptides. We then developed a high-throughput flow cytometry based screening assay to compare binding of selected peptides as displayed on-phage to IL-23 alanine variants displayed on yeast. Comparison of the binding of the peptides to wild-type versus alanine variants of IL-23 on yeast resulted in successful binning of the peptides as displayed on-phage based on binding region(s). This unique approach enabled us to reliably characterize peptides based on binding region(s) in a quick and economical manner. In this study, we also describe how to deplete libraries of peptides that interact with non-functional binding regions on the target using FACS (Fluorescence-Activated Cell Sorting). As a result, peptides against specific binding regions can be readily identified. This approach has two significant applications: it can be utilized for binning of the peptides based on binding region(s) and for depleting peptide libraries from background binders.

## Materials & methods

### Materials

Glucose agar plates 10 cm CM minus Trp/Ura from Teknova (C3260), Streakers wooden sticks from Biolog (3026), CM galactose broth Trp^-^/Ura^-^ from Teknova (C9130), CM glucose broth Trp^-^/Ura^-^ from Teknova (C8240), vented 50 mL conical flasks form TRP (87050), 96 well U-Bottom plates from Falcon (353077), 96 well plate 2 mL PP from Thomson Instrument company (931130), Blocker Casein in PBS from Thermo (QD216041), 2xYT from Teknova (2Y1080), AF647 anti-M-13 conjugated in-house according to manufacturer recommendation, Anti-V5 antibody from Invitrogen (46–1157), FITC-anti mouse IgG2a form Biolegend (407106), PE-Goat anti-mouse IgG2b from Southern Biotech (1090-09S), Human IL-12/IL-23 P40 Mab Clone1645 from R&D Systems (Mab 6091–500). Peptide synthesis was outsourced to CPC Scientific. Avi-tagged IL-23 and FLAG-tagged IL-23R were expressed and purified in house following standard Molecular Biology procedures. Alpha-screen Streptavidin Donor beads (#509048876) and AlphaLISA® Acceptor beads conjugated to anti-FLAG antibody (#AL112M) were purchased from Perkin Elmer. ProxiPlate-384 Plus plates (#6008289) for AlphaLISA assays were also obtained from Perkin Elmer. PathHunter® U2OS IL-23R/IL-12RB1 Dimerization Cell Line was purchased from DiscoverX (#93-1007C3). 96-well half area plates used in the dimerization assay were from Costar (#3688).

### Phage display library construction, selection, and screening of hits

Peptide phage libraries were generated using the previously described method [14]. To deplete libraries from streptavidin background and p40 subunit binders, libraries were screened against them prior to selection against IL-23. First round of selection was comprised of two rounds of negative selections against neutravidin-coated Beads and IL-12. Number of negative selections was increased at the subsequent rounds of selection. Phage was added to either IL-12 captured on neutravidin-coated beads or neutravidin-coated beads. After spinning down the mix, supernatant containing phage was removed to either repeat the negative selection or it was incubated with IL-23 to allow binding to the target (positive selection). The complex of IL-23 and phage was pulled down using neutravidin-coated beads. IL-23 bound phage was eluted by 100 mM of triethylamine and was immediately neutralized by 1 M Tris/HCl pH 6.8. After three rounds of selection, phage was screened against IL-23, IL-12, and background neutravidin-coated plates as described previously using filter-lift and phage-ELISAs [15].

### Surface Plasmon Resonance (SPR)

SPR (Biacore T200, GE Healthcare) was used to determine binding constants of the peptides against IL-23 and their competitiveness to IL-23R. All SPR experiments were performed at 25°C with flow rate of 50 μl/min. Assay buffer used in direct binding experiment was 10 mM HEPES pH 7.5, 150 mM NaCl, 0.005% P20, and 3% DMSO. Assay buffer used in competition experiment was 10 mM HEPES pH 7.5, 150 mM NaCl, 0.005% P20, and 1% BSA. All peptides were reconstituted first in DMSO as 10 mM stocks and then diluted into assay buffer. The proteins used in direct binding and competition SPR are described in S1 Table in S1 File. For direct binding of peptides against IL-23, 2000–9000 RU of IL-23-AVI was captured to streptavidin sensor chips (GE Healthcare, Series S Sensor Chip SA, BR-1005-31). Dilution series with 3-fold increment were prepared for each peptide. Initially, all peptides were tested up to 30 μM. Concentration range for some were then lowered accordingly based on affinity or nuisance behavior at high concentrations. SPR “multi-cycle” type was used for peptides 23–644, 23–437, 23–441, 23–446, and 23–447. Each cycle consisted of 30s of analyte injection and then 60s of buffer flow to monitor dissociation. “Single cycle kinetics” (SCK) type was used for 23–652 due to their long dissociation half-lives. Each SCK cycle consisted of five 60s injections of sample in increasing concentration with a final 600s of dissociation. To study IL-23 and IL-23R interaction, 200–1000 RU of IL-23-AVI and IL-23R-Fc-AVI were captured to streptavidin sensor chip. Dilution series with 3-fold increment of IL-23R-FLAG-HIS and IL-23-HIS were run in SCK cycle type as described above except the final dissociation time were 60-300s. Sensorgrams from the entire titration series for each sample were fitted globally to extract binding kinetics and affinity using Biacore T200 Evaluation Software 2.0 (GE Healthcare) according to a 1:1 binding model. To identify IL-23 binders that can block receptor binding, a competition SPR was established. In each SPR cycle, fresh IL-23R was capture (<1000 RU) by injecting 10 μg/ml of IL-23R-Fc-AVI to a Protein-A flow cell at 10 μl/min flow rate for 30s. Analyte consisted of a mixture of 20 nM of IL-23 protein and a single concentration of peptide was then injected for 30s at 50 μl/min to observe IL-23 binding. Finally, the Protein-A surface was regenerated by 10 mM Glycine pH 1.5 for 30s at 30 μl/min. IL-23 at 20 nM without peptide, “IL-23 only”, was used as control to quantify IL-23 binding without blockage; while “cold” IL-23R was used as a positive control for blockage.

### IL-23:IL-23R protein-protein interaction assay: AlphaLISA and TR-FRET

For AlphaLISA assay, peptides at concentration of 10 mM were dissolved in DMSO and serial 3-fold dilutions were carried out using acoustic technology, dispensing peptides directly into the assay plate (ProxiPlate-384 Plus). The rest of the reagents were dissolved in assay buffer containing 25 mM HEPES pH 7.4, 150 mM NaCl, 0.02% Tween 20 and 0.1% BSA. IL-23-AVI was pre-incubated with peptide at the indicated concentrations for 30 min at room temperature. Then, FLAG-tagged IL-23R was added to the assay mix and incubated at room temperature for 1 hour. This was followed by addition of Streptavidin-coated donor beads and acceptor beads conjugated to anti-FLAG antibody. Final assay concentration of IL-23-AVI and FLAG-IL-23R were 1.5 nM and 3 nM, respectively. The assay plate was incubated for one additional hour at RT and read in an Envision plate reader. For TR-FRET assay, tested compounds were serially diluted in DMSO using an Echo acoustic and dispensed directly into the final assay plate. Final concentration of DMSO in the assay was 1%. Assays were performed by preincubation of the diluted compounds with IL-23R-FLAG for one hour at room temperature in assay buffer containing 25 mM HEPES, 150 mM NaCl, 0.02% Tween 20, 0.1% BSA; pH 7.4. Next, IL-23-AVI was added to the assay mix and incubated for 1 hour at room temperature. Finally, the detection reagents, Europium-labeled streptavidin and Anti-FLAG IgG conjugated to SureLight–Allophycocyanin were added and incubated overnight at room temperature. Then, assay microplates were read and values were collected as the ratio between fluorescence emission at 665 nm and 615 nm (665/615). The final values were expressed as a percentage of the DMSO treated controls. IC_50_ values were calculated by fitting the normalized data to a four parameter logistic equation.

### Generation of IL-23 Alanine-scanning mutagenesis using yeast display technology

Correct assembly of IL-23 heterodimer requires co-expression and co-localization of p19 and p40 subunits on the surface of *Saccoromyces cerevisiae* yeast cells. To accomplish this, we cloned the p40 gene into a soluble-expression plasmid (proprietary pYKY vector) containing a uracil selection marker under the control of a constitutive promotor. The p19 subunit was cloned into a surface-display plasmid (proprietary pTS6 vector) with a tryptophan selection marker under the control of a galactose-inducible promoter. The p19 subunit is expressed as an Aga1 fusion protein with a modified GPI anchor with V5 tag, which was used to confirm protein expression. The display vector was designed to allow alanine mutants of the p19 subunit to be constructed using Kunkel mutagenesis. Specific reverse compliment oligonucleotides (from IDT) encoding alanine at each position were annealed to a single-strand uracil-containing phagemid vector with wild-type p19. Complementary strand was synthesized using T4 DNA polymerase (NEB) and the resulting duplex was treated with T4 DNA ligase (Roche). The constructs were transformed into competent *E*. *coli* cells (Invitrogen), grown under selection conditions, and confirmed for successful integration by sequence analysis. The constructs were transformed into naïve yeast cultures using lithium acetate/ssDNA/PEG method. BJ5464 cells (ATCC) are grown on YPD (Teknova) plates for 2–3 days following an initial thaw and plating. A small patch of cells is used to inoculate 10 ml of YPD broth (Teknova) in a vented conical tube (Corning) and these cells are grown at 30°C at 200 rpm in an incubator shaker overnight. Cell density was measured with a spectrophotometer and diluted to OD_600_ of 0.2 in YPD (in a volume determined for the number of transformations needed) in vented Erlenmeyer flasks (Corning) and grown at 30°C for ~4 hrs until the OD_600_ of 0.8–1.2 is reached. The cells are centrifuged gently, washed once with water, and resuspended at approximately 1e8 cells in 50 μl in individual Eppendorf tubes. Following the removal of the water by brief centrifugation, the following components are added to cells in the following order: 240 μl PEG3500 (at 50%w/v; Sigma), 36 μl 1 M lithium acetate (Sigma), 50 μl ssDNA (1 mg/ml; denatured), 36 μl of plasmid DNA plus d/d water. The optimal amounts of p19 display vector and p40 secretion vector were determined to be 800 ng and 200 ng, respectively. The cells with the transformation mix are vortexed vigorously for one minute then incubated at 42°C for one hour with an occasional gentle mix. The transfection mix is removed following centrifugation, and the cells are resuspended in water and plated out onto CM/glucose plates without uracil or tryptophan (Teknova). The plates are sealed and grown for 3 to 4 days in a 30°C incubator, thereafter stored at 4°C. Individual colonies are picked into 10 ml of CM glucose Trp^-^/Ura^-^ broth (Teknova) in vented conical tubes and grown overnight at 30°C at 200 rpm in an incubator shaker. The following day approximately 3 ml of this growth stage culture is collected and following media removal, resuspended in 10 ml CM galactose Trp^-^/Ura^-^ media (Teknova). These cultures are grown at 20°C at 200 rpm in an incubator shaker overnight. Typically 100 μl of the induced cultures are added to each well of a deep-well block (Thomson) for staining of membrane associated proteins and specific antigens.

### Display of wild-type and alanine mutants of IL-23 on yeast

IL-23 protein expression on the yeast surface was assessed by flow cytometry by antibodies specific for the p40 subunit (R&D Systems) and V5 tag (Invitrogen) using Fortessa X-20. PE or FITC-conjugated secondary antibodies were used for the p40 and V5 (Jackson Immunochemicals), respectively. Ten thousand events were collected for each alanine-mutant clone at a cell density of ~1e6 cells/ml. Intact yeast cells were identified using forward and side scatter gating parameters followed by identification of p40 expressing cells. Clones with very low or no p40 subunit levels were excluded from the analysis. Binding of the p19 antibody to the p40 positive cells was directly measured through an AlexaFluor647 label. FlowJo software was used to calculate the median fluorescence intensity for the FITC (V5) and AlexaFluor647 (p19) channels.

### Binding of peptide-phage to IL-23 variants displayed on yeast

A total of 1e9 yeast cells were induced, stained, and aliquoted into 96 well 2 mL deep blocks. This was followed by addition of phage, V5 and hu IL-12/IL-23 P40 antibodies for staining. This mix was incubated for 1 hour at RT. Following wash with Casein in PBS, AF647 conjugated anti M-13, FITC-anti mouse IgG2a, and PE-Goat anti-mouse IgG2b were added and incubated for 1 hour at RT. Mix was washed again casein in PBS and re-suspended in casein in PBS in 96 well U-Bottom plates. Plates were read on Fortessa X-20.

### HDX-MS

Hydrogen-deuterium exchange/mass spectrometry (HDX/MS) experiments were performed using a Waters Corporation integrated HDX/MS platform, including a LEAP HDX robotic liquid handling system, a nanoACQUITY UPLC, a HDX chilled column switching manager, and a Xevo G2/xs mass spectrometer. The mobile phases used consisted of water (A) and acetonitrile (B), each containing 0.2% formic acid. The complex of hu IL-23 with a peptide was prepared at the molar ratio of 1:5 (hu IL-23 to Peptide) in 10 mM sodium phosphate buffer, pH 7.4 containing 150 mM NaCl (1xPBS buffer), where the final concentration of hu IL-23 was 1.8 mg/ml in the complex. The free hu IL-23 (1.8 mg/ml) and the complex of hu IL-23:Peptide were placed into the labeling tray as 5 μl aliquots; to initiate exchange, each aliquot was diluted in 55 μl of D_2_O containing 0.1xPBS buffer, and incubated at 15˚C for various amounts of time. The labeling reactions were quenched for 2 minutes at 1˚C with an equal volume of 320 mM TCEP, 100 mM phosphate (pH 2.5). The quenched exchange reactions were immediately injected into a 50 μl sample loop, followed by on-line pepsin digestion using a Waters BEH Enzymate pepsin column at 14˚C with mobile phase A at the flow rate of 100 μL/min for 3 minutes. The resulting peptic peptides were trapped on a Waters BEH Vanguard Pre-column at 0.5˚C and chromatographically separated using a Waters Acquity UPLC BEH C18 analytical column at 0.5˚C at flow rate of 50 μl/min and a gradient of 3%–85% mobile phase B over 10 minutes. The sample was directed into the Xevo G2/xs mass spectrometer for mass analysis. The Xevo G2/xs was calibrated with Glu-fibrinopeptide prior to use. Mass spectra were acquired over the m/z range of 255 to 1950 in ESI+ mode, with the lock mass *m/z* of 556.2771 from Leucine Enkephalin. The peptic peptides were initially identified by Waters ProteinLynx Global Server 2.5 using a combination of accurate mass and fragmental ions (MS^E^ mode). The resulting peptide list was imported to Waters DynamX 3.0 software, where the relative deuterium incorporation for each peptide was determined by processing the MS data for deuterated samples along with the undeuterated control. The free and bound states of hu IL-23 were compared for deuterium incorporation differences.

HDX-MS experiments of IL-23 with blocking peptides were performed as described elsewhere [16]. For each experiment hu IL-23 was incubated with peptide at a 1:5 molar ratio in 50 mM TRIS-HCl buffer (adjusted to pH 7.5) containing 50 mM NaCl before deuterium labeling. The on-exchange reaction was initiated by a 5-fold dilution of a 10 μM protein stock solution (in the presence or absence of peptide) in the corresponding D_2_O buffer at room temperature. Thus, samples were prepared with three replicates of the following exchange times; Dmin, 10s, 30s, 90s, 270s, 810s, 2430s, 7290s and Dmax. At each time point, HDX reactions were quenched with a solution of 320 mM of TCEP, 100 mM phosphate (pH 2.5) followed by online pepsin digestion and desalting through a C_8_ trap column. The resulting proteolytic peptide mixtures were separated by reversed phase liquid chromatography on an analytical C_18_ column and eluted into the mass spectrometer (Q-Exactive, Thermo Scientific). HDX-MS data were processed with HDX Workbench software [17].

### FACS

Ten million yeast cells displaying IL-23 wild-type or its alanine variants were induced, washed and aliquoted into 96-well 2 ml deep blocks. 1e12 pfu of phage library was added to the yeast cells and allowed to incubate for 1 hour at room temperature. The yeast was then washed with casein in PBS to remove any non-binding phage. Anti-M13 monoclonal antibody (GE Life Sciences, cat. No. 27942001, DISCONTINUED) conjugated to AF647 (performed in-house) was added and allowed to incubate for 1 hour at room temperature. The samples were washed again with casein in PBS and re-suspended in casein PBS in 96-well u-bottom plates. Propidium iodide staining solution (BD Biosciences, cat. No. 556463) was added per manufacture protocol for live cell staining. Set-up and sorting of the AF647 positive events was performed on the BD FACSAriaIII. Yeast cells + anti-M13-AF647 was used to identify/gate the background population and non-specific binding. The collected AF647 positive events were then processed by eluting the phage bound to the yeast cells using triethylamine and neutralizing with Tris-HCl pH 6.8. The eluted phage was titered and random plaques were picked for sequencing.

## Results

### Selection of phage libraries against IL-23

Different cyclic peptide libraries consisting of 12, 15 and 18 random amino acids and one linear 15-mer library were selected against IL-23. The cyclic libraries contained different numbers of random amino acids in the loop and the flanking regions next to cysteines (S2 Table in S1 File). To increase chances of finding peptides specific to the p19 subunit of IL-23, we first removed background and p40 subunit binders by selecting the libraries against neutravidin and Avi-tagged IL-12. IL-23 and IL-12 share a common subunit, p40, therefore library selection against IL-12 should remove the p40 binders. Depleted libraries were then selected against 100 nM of IL-23-AVI. As a result, a gradual enrichment of p19 binders was observed in the selected pool as the selection progressed. After three rounds of selection, phage pool was deconvoluted by filter-lift and single point ELISA [15]. Sequence analysis of specific p19 binders indicated that more than 40% of clones share a common motif comprised of phenylalanine-Glycine-Leucine/Threonine (FGL/T).

### Construction, display, and validation of IL-23 and its alanine variants on yeast

**Fig 1:**
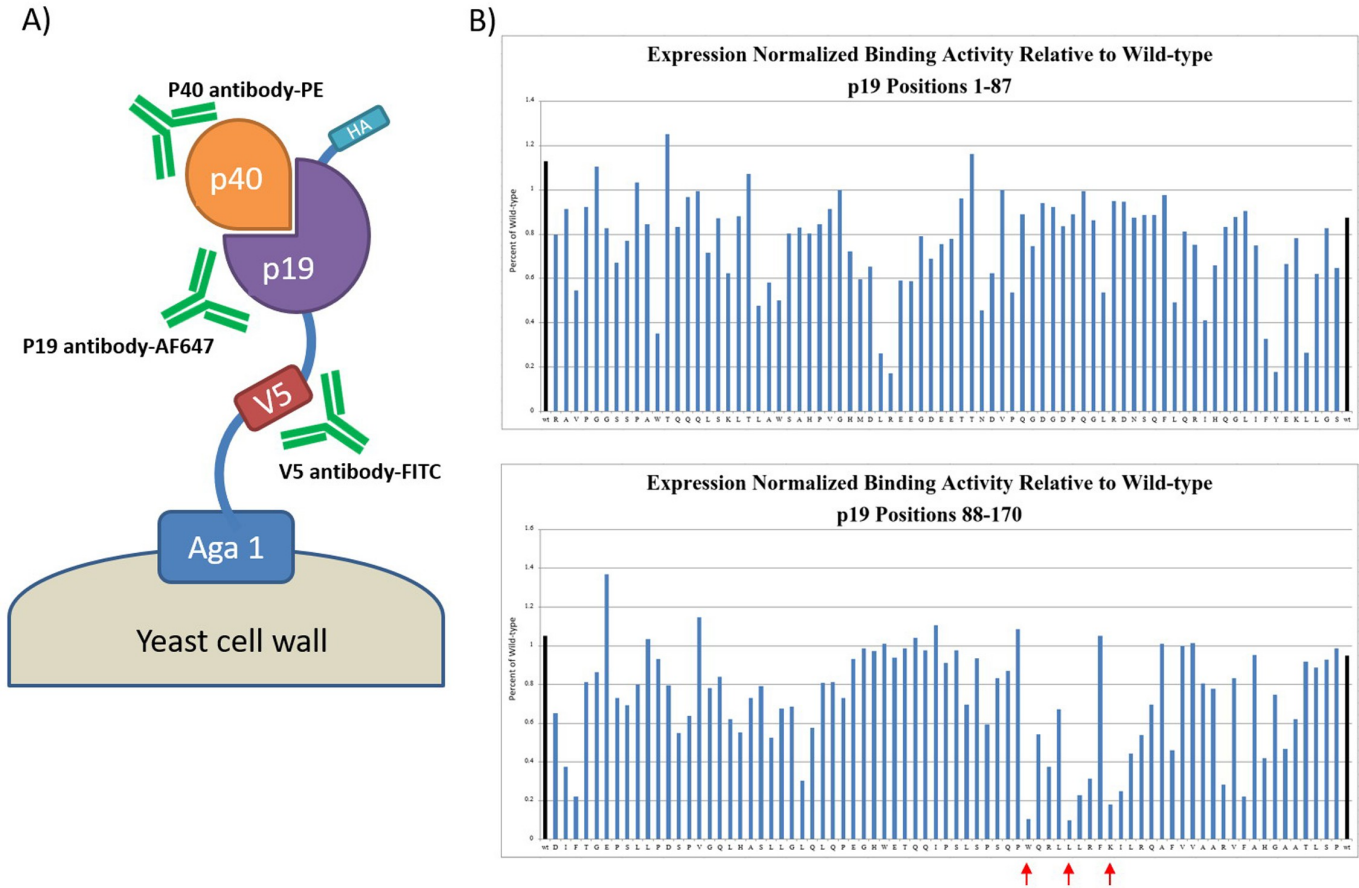
Display of IL-23 and p19 alanine variants on yeast. A) P19 subunit was anchored on yeast surface by fusion to vial V5-tag. P40 was expressed as soluble protein. Antibodies against p19, p40, and V5-tag were used to isolate yeast cells with correct assembly of IL-23 heteromer. B) Normalized binding of IL-23R to wild-type IL-23 and alanine variants of p19 displayed on yeast (n = 1). Binding of wild-type IL-23 is shown in black bar and binding of W137A, L141A, and K145A are indicated by red arrows.

We asked if we could integrate phage and yeast display platforms to create a technique to bin our peptides on-phage based on their binding region on IL-23. For this purpose, we displayed wild-type IL-23 and alanine variants of p19 subunit on yeast to assess their binding to peptides on-phage using flow cytometry (Fig 1A). P19 was anchored on yeast surface by fusion to Aga1 using a modified GPI anchor. A V5-tag was inserted between p19 and Aga1 to enable quantification of p19 displayed on yeast surface. P40 was expressed as soluble protein and was to assemble with the anchored p19 on the yeast surface. To ensure that protein is properly folded on yeast and is free of aberrant post-translational modifications, we utilized specific antibodies to p19 (Alexa Fluor 647), p40 (PE), V5-tag (FITC), and fluorescently labeled IL-23R to isolate yeast cells with correct assembly of IL-23. First, we confirmed p40 display on yeast cells and discarded cells with low or no p40 display. We normalized p19 display level on p40 positive yeast cells by determining the ratio of median fluorescence signal from AF647 (against p19) to FITC (against V5-tag) (Fig 1A). Fluorescently labeled IL-23R was used to assess functional display of IL-23 on yeast cells. IL-23R interacted strongly to wild-type and most of ala-mutants, except for clones with alanine mutation at residues 137, 141, and 145 of p19 (Fig 1B). These residues, in particular W137, have previously shown to be major sites for receptor binding [18, 19].

### Feasibility assessment for Integration of yeast and phage display platforms

**Fig 2:**
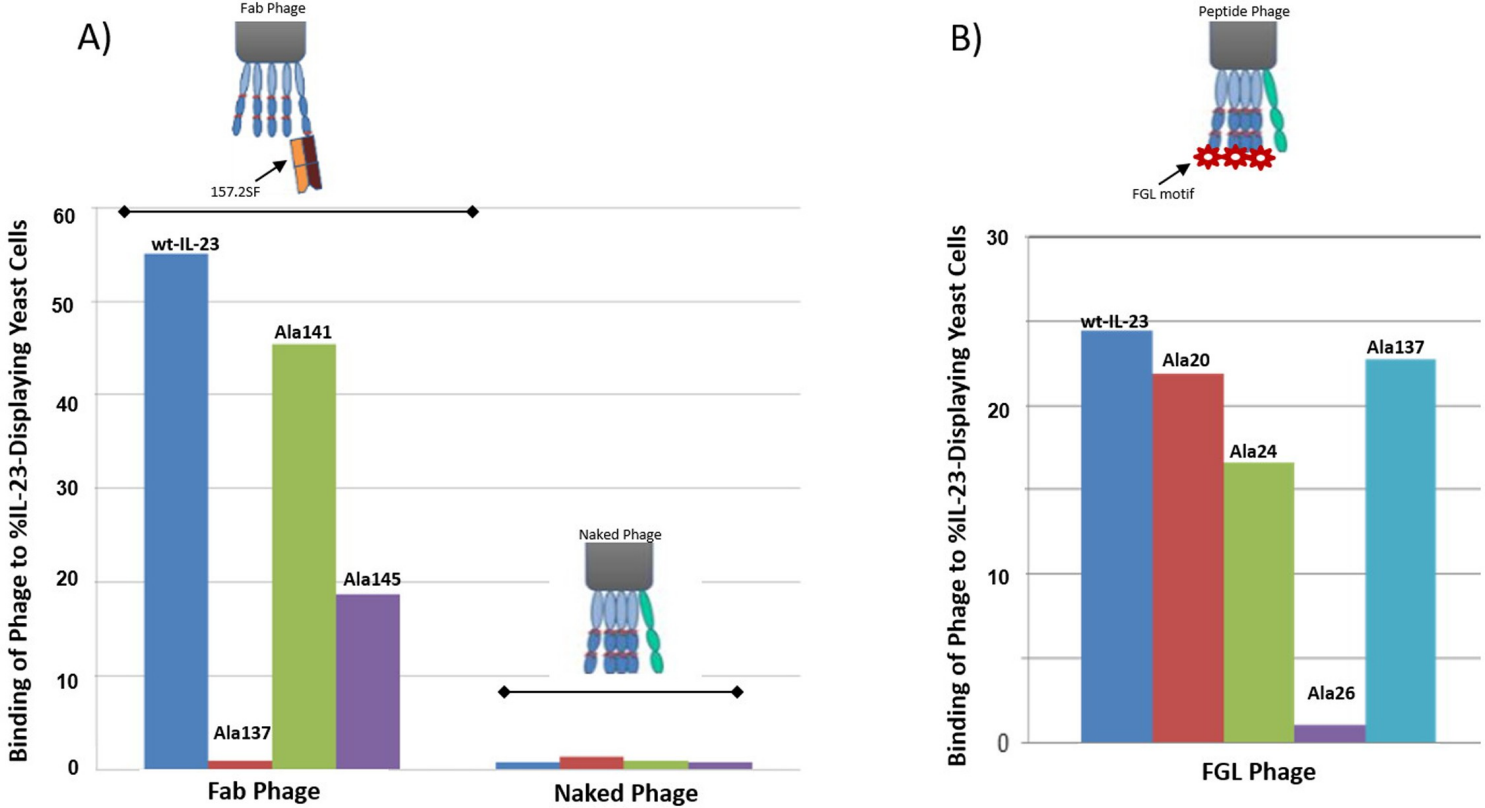
Binding of phage to IL-23 and its alanine variants of p19. A) Binding of Fab 157.2 displayed on-phage to wild-type and W137A, L141A, and K145A variants of p19 displayed on yeast. Binding of phage to %IL-23-Displaying Yeast Cells refers to mean fluorescent intensity derived from M13-AF647 antibody after the fraction of IL-23 displaying yeast cells were identified. IL-23 display was confirmed by V5-tag and p40 antibodies. Binding of phage to IL-23 was detected using anti-M13-AF647. IL-23 displaying yeast cells in the presence of M13 antibody was used for gating of all other samples. Fab-phage interacts strongly with wild-type IL-23 and does not bind to W137A variant. Naked phage does not bind to wild-type or alanine variants of p19 (n = 5). B) FGL-containing peptide phage binds to wild-type and all the alanine variants of p19 except for W26A. FGL-phage binds at reduced levels to L24A (n = 2).

To test the feasibility of our approach, we decided to display Fab portion of an internally discovered neutralizing antibody called 157.2 on-phage. The antibody bound to recombinant IL-23 with 1 nM affinity and competed with IL-23R for binding to IL-23. After confirming specific binding of Fab-phage to recombinant IL-23 in a titer dependent phage ELISA, we looked at binding of 157.2 Fab-phage to wild-type IL-23 as well as to W137, L141, and K145 alanine mutants of IL-23 on yeast that had abrogated binding of the receptor [19–21]. We used naked phage that is devoid of any display as negative control to make sure that the phage does not bind to yeast. Fab-phage bound to wild-type IL-23 and to all the p19 alanine mutants except for Ala137 (Fig 2A). 157.2 Fab-phage also bound much weaker to Ala145 compared to wild-type IL-23. Naked phage did not bind to wild-type or any of the alanine mutants of IL-23 (Fig 2A). Next, we asked if we could utilize this approach to categorize peptides on-phage based on their binding region.

### Binning of FGL-motif containing peptide-phage

Binding of two FGL containing peptides on-phage, 23–644 and 23–652 to IL-23 was confirmed in a titer dependent phage ELISA. Neither one of the peptides bound to IL-12. We next tested these two peptides for binding to wild-type and alanine variants of IL-23 on yeast. We included naked phage as a control in the assay. As before, naked phage did not bind to any of the IL-23 clones displayed on yeast. Fab-phage bound strongly to wild-type IL-23 and not to W137 and K145 ala-mutants of p19. Both FGL-phage bound to wild-type IL-23 and its ala-variants including Ala137, except for Ala26 (Fig 2B). Both peptides also showed reduced binding to Ala24. This suggested that the peptides bind to IL-23 at a region distinct from IL-23R and that they should not block interaction of IL-23 with its receptor. To confirm our finding, we chemically synthesized 23–644 and 23–652 for further analysis including determination of their binding region by HDX.

### HDX-MS: IL-23 free and bound states

**Fig 3:**
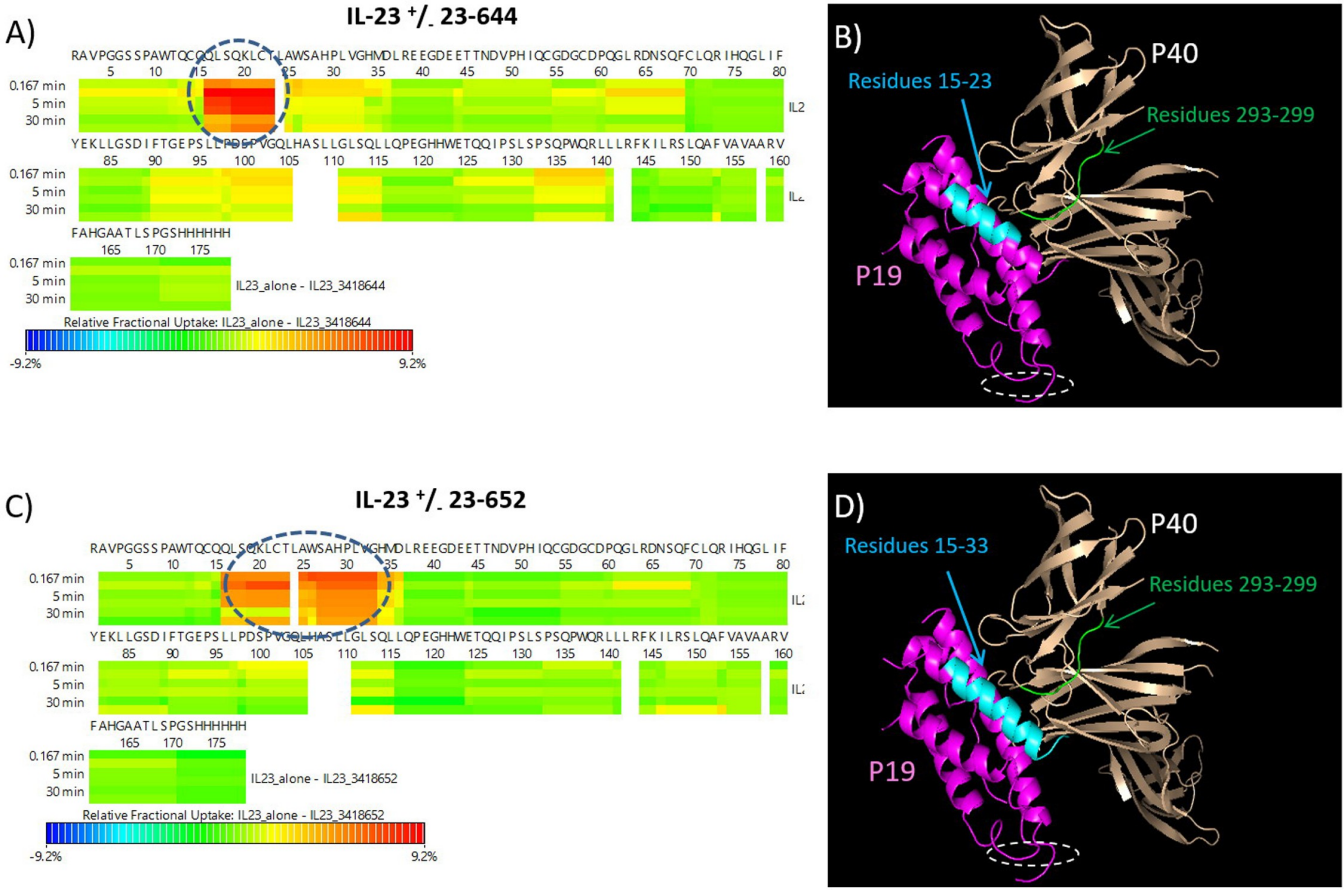
Deuterium uptake plots of IL-23 in the presence and absence of peptides determined by HDX MS. A) Regions on IL-23 with differences in deuterium uptake is shown for A) 23–644 and C) 23–652. The binding region for 23–644 and 23–652 are linear and includes residue 15 to 23 (QQLSQKLCT) and 15 to 33 (QQLSQKLCTLAWSAHPLVG) on p19, respectively. These regions are indicated in dashed blue circles. No significant deuterium uptake difference was observed in p40 subunit, except for possible binding/blockage or structural changes at residues 293–299. This region is spatially proximal to peptide binding site on p19. Crystal structure 3DUH was used to show a surface view of IL-23 that is involved in peptide binding is shown for B) 23–644 and D) 23–652. IL-23R binding site is shown in white dashed circle.

A detailed description of IL-23 dynamic behavior in solution is described in the supporting information. Deuterium incorporation of IL-23 alone was compared to the deuterium incorporation of the IL-23-FGL-containing-peptide complexes. Two FGL-containing peptides (23–644 and 23–652) were incubated with IL-23 for 1 hour prior to the start of the HDX experiment. The deuterium uptake measurements over time for each state, bound and unbound, were compared to investigate differences in rate of exchange. We observed protection from deuterium uptake in the p19 subunit and minor protection in the p40 subunit for both peptides (Fig 3 and S1 Fig in S1 File). A decreased level of deuterium uptake in p19 Helix A at amino acids 15–23 (QQLSQKLCT) was observed for peptide 23–644. Small differences in exchange signifying minor blockage or a structural change from amino acids 24–33 (LAWSAHPLVG) were also noted for peptide 23–644. Binding of peptide 23–644 induced two changes in p40 subunit: a decreased deuterium exchange at the D1 domain of the p40 subunit at amino acids 293–299 (YSSSWSE) (Fig 3A and 3B and S1A and S1B Fig in S1 File) and small structural changes in its all three of its domains. HDX-MS results for peptide 23–652 showed decreased deuterium uptake in Helix A at amino acids 15–33 (QQLSQKLCTLAWSAHPLVG) and decreased deuterium exchange in the D1 region of the p40 subunit at amino acids 293–299 (YSSSWSE). There were no structural changes in the D2 or D3 domains of the p40 subunit upon binding to the peptide (Fig 3C and 3D and S1C and S1D Fig in S1 File). A crystal structure of IL-23 in complex with 23–652 was determined and confirmed the HDX result. The co-crystal structure revealed that all interactions of the peptide 23–652 with IL-23 are confined to p19 helix A, a small section of the p19 AB loop, and D3 of p40 (S2 Fig and S3 Table in S1 File). In particular Trp26 (p19) was flipped out relative to the apo structures [18, 21] to pick up an interaction with Phe7 of 23–652 (S2D Fig in S1 File). HDX and co-crystal structure results confirmed the earlier finding that W26 is in fact a hotspot residue for binding of peptides 23–644 and 23–652, since neither one of the peptides retained binding to the W26A mutant of IL-23.

### Identification of IL-23 blocking peptides as displayed on-phage

**Fig 4:**
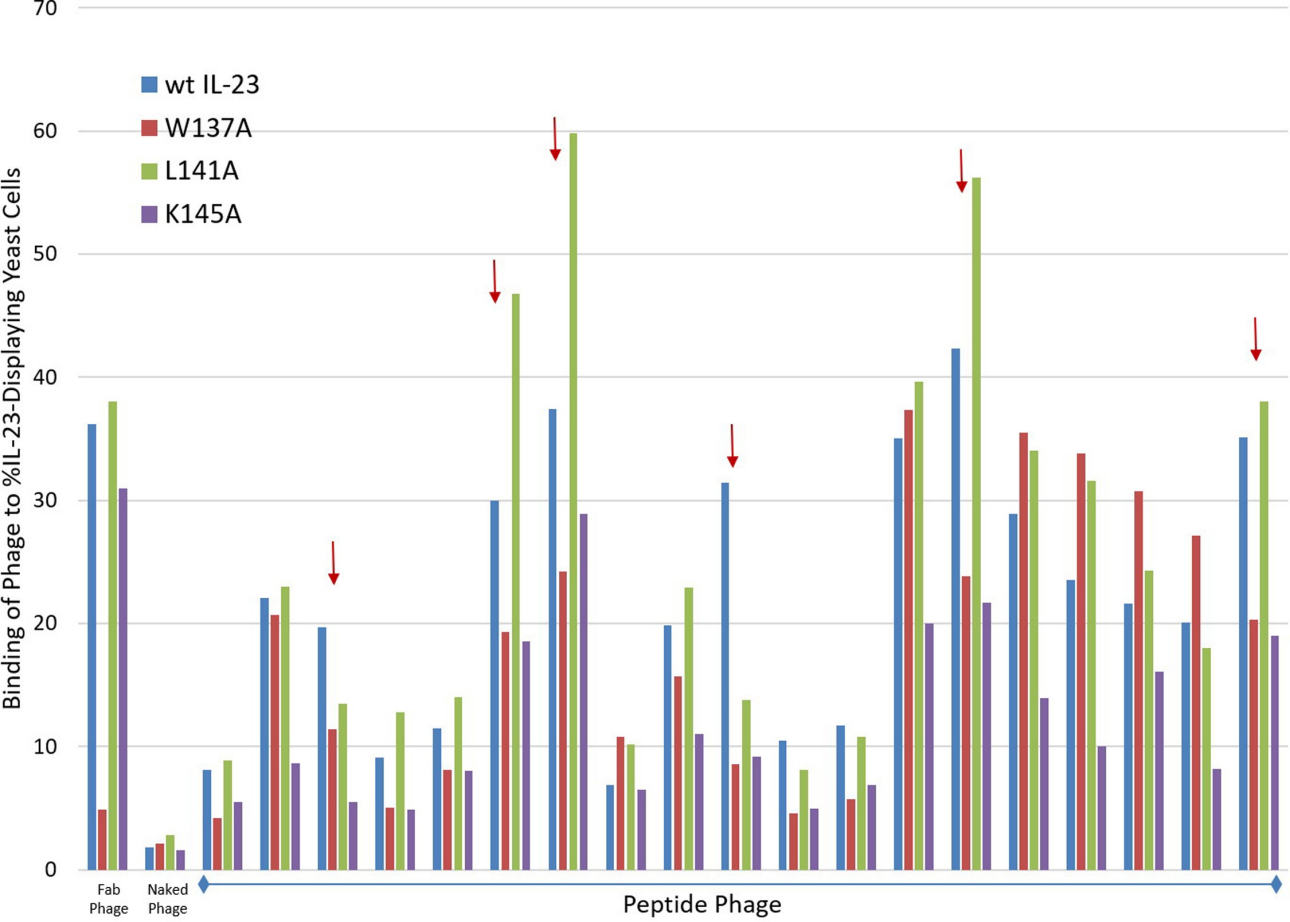
Binding of phage to IL-23 and W137A, L141A, and K145A variants. Binding of peptide-phage to wild-type IL-23 was compared to three p19 alanine variants including W137A, L141A, and K145A using flow cytometry. Peptides with less than 10% positive signal (chosen arbitrarily) were eliminated. Peptides with considerable reduction in binding to W137A variant compare to wild type were considered as potential blockers and are marked by an arrow (n = 2). Fab and naked phage was used as controls.

We next asked if we could apply the same approach to identify IL-23 blocking peptides as displayed on-phage. For this purpose, we used the deconvoluted phage pool from round three. We utilized single point ELISA to identify peptides on-phage that bind to recombinant IL-23. Peptides that bound to either IL-12 or to background surfaces were eliminated and IL-23 binders were sequenced. As expected, majority of the binders contained the FGL/T motif, suggesting that the percentage of blockers might be lower than expected. We next re-arrayed the binders in 96-well deep blocks to exclude redundant sequences and FGL/T containing peptides. The re-arrayed phage was amplified and tested for binding to wild-type and three IL-23 alanine mutants (W137A, L141A, and K145A) displayed on yeast using flow cytometry. The three alanine mutants were chosen based on the earlier result obtained from screening of IL-23 mutants on yeast against recombinant IL-23R (Fig 1B) as well as published data [20, 21]. We assumed that the peptide-phage that binds comparably to wild-type IL-23 and the three alanine variants should not block interaction of IL-23 with its receptor. On the contrary, if the peptide binds strongly to wild-type IL-23 and not to any one (or all) of the three alanine mutants, the peptide should be marked as a potential blocker (Fig 4). To check our hypothesis, we tested 212 unique peptides displayed on-phage against the four different IL-23 constructs on yeast. As before, we used Fab-phage and naked phage as controls. We identified less than twenty peptides that bound to wild-type IL-23 much stronger than to either one of the alanine variants (W137A, L141A, and K145A) some of which are shown in Fig 4. These peptides were considered as potential blockers since they bound to wild-type IL-23 stronger than to alanine variants of IL-23 at the receptor binding site. Based on sequence homology among hits, five peptides (23–437, 23–441, 23–443, 23–446, and 23–447) were chemically synthesized to confirm their binding and functional activity. We also included free peptides 23–644 and 23–652 in that analysis.

### SPR analysis of free peptides: Binding and competition

Affinity of IL-23 to its receptor was determined with either the ligand or the receptor immobilized on the surface of the SPR chip. Affinities obtained from either format were comparable with only some differences observed in the kinetics (Table 1). This variance could be due to oligomeric states of the two IL-23R proteins (dimer for IL-23R-Fc-AVI and monomer for IL-23R-FLAG-HIS) or steric differences due to immobilization. All peptides except 23–447 could reach or approach saturation and fit well to 1:1 binding model (S3 Fig in S1 File). Peptide 23–447 had low affinity and could not be dosed to saturation. Therefore, the kinetics and affinity for 23–447 should be considered as an estimate. Peptides 23–441 and 23–644 exhibited fast on and off secondary binding components even with solvent correction. This was treated as bulk reflective index (RI) difference between sample and running buffer during fitting. The slower main binding event appeared saturable and could be fitted well to a 1:1 binding model. Peptide 23–446 had fast on rate that could be approaching the detection limit of SPR. Therefore, a much lower density sensor might improve the accuracy of the reporting kinetics. Peptide 23–443 did not retain its binding to IL-23 as a free peptide.

**Table 1:** Peptide sequences and their characterizations by SPR (n = 1).

	Sequence	ka (1/Ms)	kd (1/s)	KD (M)	Competition SPR	
					%Inhibition	Concentration tested (μM)
IL-23R		1.70E+05	1.51E-03	8.92E-09	98.0	0.1
23–652	DTLTKSFCYFGTWCQMYGST	3.66E+04	2.63E-03	7.18E-08	0.4	3
23–644	HVPDPCWFGLLCDVLNS	4.93E+04	1.29E-01	2.62E-06	Not done	-
23–437	FTKEIDSWWLSLMSS	1.78E+05	1.86E+00	1.05E-05	57.6%	30
23–441	QKITQCVYIIWTYPCLLNDV	1.90E+05	1.74E-01	9.11E-07	100.0%	30
23–446	GLRERCVRNYGHEFCQNWYW	3.88E+06	8.63E-02	2.23E-08	79.1%	3
23–447	LECLYYLRWKVCDN	4.83E+04	1.33E+00	2.75E-05	78.7%	30
23–443	LLQTLCQMTSDARTCMLWSI	-	-	*NB	-	-

* NB: not binding. Chemically synthesized peptide did not retain binding as free peptide.

**Fig 5:**
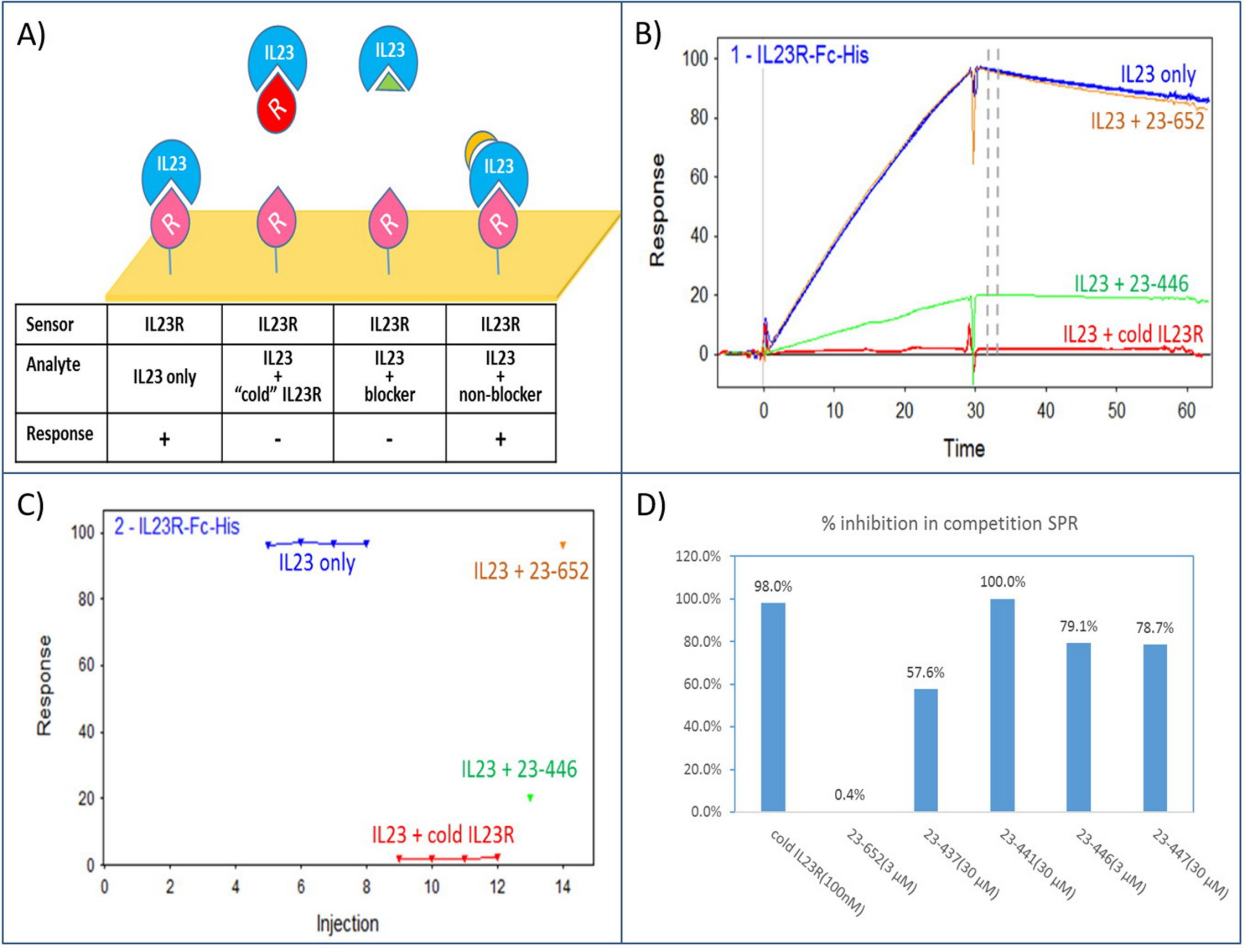
Competition SPR to identify IL-23R blockers. A) The cartoon principle of the competition SPR is shown. Pink “R” is IL23R-Fc-AVI receptor protein captured by Protein A chip; red “R” is IL23R-FLAG-HIS receptor protein as positive control for IL23R blockers; blue “IL23” is IL23-HIS protein; green triangle represents IL23R blocking peptides; and orange crescent represents non-blocking peptides. B) An example of the competition SPR experiment with “IL23 only” (blue); mixture of IL23 + IL23R blocking peptide (23–446, green), non-blocking peptide (23–652, orange), and positive control (cold IL23R, red). “IL23 only” and “IL23 plus cold IL23R” were run in quadruplicates and their sensorgrams were superimposed. Grey dash lines indicate the location where the response level for each sample was measured. C) The response level of each samples in (B) are shown (n = 1). D) A summary of a % Inhibition indicated by competition SPR. The concentrations listed in parenthesis were the concentrations of the peptides used in the competition (n = 1).

The design of competition SPR was based on a knockdown assay (Fig 5A). When applied simultaneously, an IL-23R blocking peptide should decrease binding of IL-23 to the surface immobilized IL-23R, while a non-blocking peptide should have no effect (Fig 5A and 5B). In this assay, IL-23R capture and regeneration was very reproducible, resulting in a very stable response of “IL-23 only” run (Fig 5B and 5C). To maximize occupancy, peptides were used at concentrations >10X KD in the competition assay. The exception were peptides 23–437 and 23–447 that were used at concentrations 3x and 1.1x above KD, respectively, due to their low affinity. Despite lower concentration, the occupancies of these two peptides against IL-23 were estimated to be higher than 50%. We decided to use IL-23 at 20 nM with short injection time of 30s. This careful choice was made to generate high enough signal window that was far from saturation of the IL-23R sensor (indicated by lack of curvature in sensorgram, Fig 5B). Therefore, after the peptides bind to IL-23, the observed binding level of IL-23 to immobilized IL-23R should be proportional to the concentration of IL-23 that has its IL-23R binding site unoccupied. Response levels of IL-23 plus peptide were measured post injection (Fig 5B) to avoid possible bulk RI difference between analyte and running buffer. Percentage inhibition (% inh) by peptides was calculated from response levels using the equation: % inh = 100*(R _IL-23 only_−R _IL-23+peptide_)/R _IL-23 only_ where R _IL-23 only_ and R _IL-23+peptide_ are response levels of “IL-23 only” control and IL-23/peptide mixtures, respectively (Table 1 and Fig 5D). Cold IL-23R achieved nearly full inhibition of IL-23 binding against IL-23R sensor. Out of five peptide-phage that were identified as potential blockers and chemically synthesized, four peptides that retained their binding to IL-23 as free peptides were confirmed as blockers (23–237, 23–441, 23–446, and 23–447). As expected FGLT-containing peptide, 23–652 was confirmed as non-blocker (Table 1).

### Confirming peptide activity by AlphaLISA and TR-FRET

We utilized two different protein-protein interaction assays, AlphaLISA and TR-FRET, to confirm the SPR result. Peptides 23–437, 23–441 and 23–447 disrupted the complex formed by IL-23 and IL-23R in AlphaLISA assay with single digit micromolar potency (Table 2). The most potent peptide, 23–446, with an IC_50_ value of 240 nM was identified as the strongest binder by SPR (Table 1), suggesting that the potency of the peptides could be improved upon affinity maturation. Potency values obtained by the AlphaLISA assay were confirmed by TR-FRET technology (Table 2). Taken together, all this data suggests that the peptides 23–437, 23–441 and 23–446, and 23–447 bind specifically to IL-23 in a binding pocket that, when occupied, will disrupt the interaction of IL-23 with its cognate receptor.

**Table 2:** Blocking activity of peptides determined by AlphaLISA and TR-FRET (n = 1).

Name	AlphaLISA (M)	TR-FRET (M)
	**IC50 (M)**	**IC50 (M)**
23–437	1.4 X 10^−6^	7.0 X 10^−6^
23–441	2.5 X 10^−6^	5.7 X 10^−6^
23–446	2.4 X 10^−7^	5.9 X 10^−7^
23–447	6.7 X 10^−6^	2.4 X 10^−5^

### HDX analysis of the blocking peptides confirms the result of binning peptides on-phage based on their binding region

**Fig 6:**
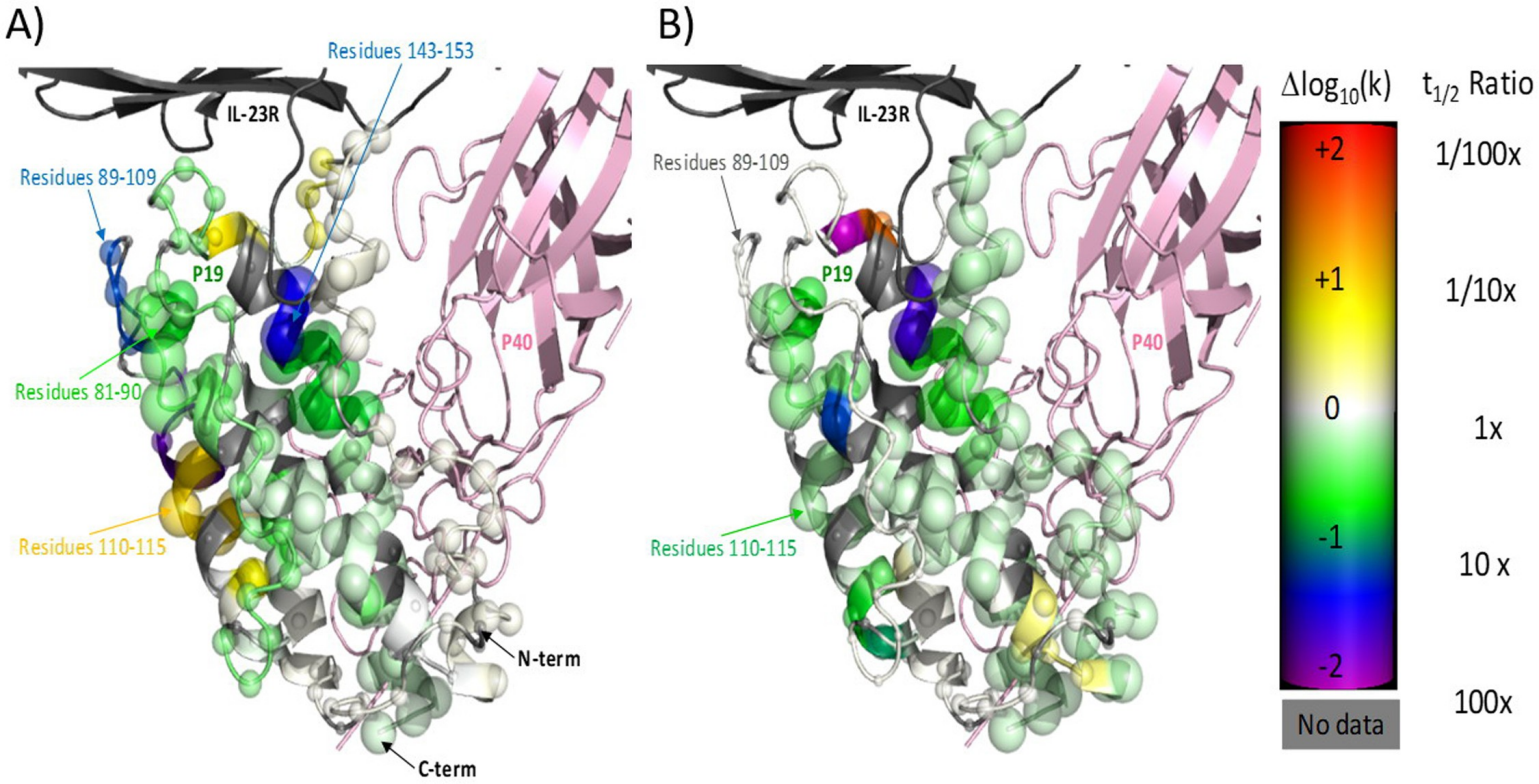
Differential HDX of IL-23 in complex with 2 peptides versus the apo state illustrated on a model of IL-23 built from 5MZV. Differential HDX of IL-23 in complex with A) 23–446 and B) 23–437. The color (generated using the pymol spectrum command) of each alpha carbon represents the magnitude of the change in calculated H-D exchange rate constant upon binding of the corresponding ligand with purple < = -2.0, blue, green, white = 0.0, yellow, orange, and red > = +2.0 log units. The size of each sphere indicates confidence that the apparent signal is truly non-zero. It is determined based upon the mean and standard deviation of the acceptable models from the Bayesian analysis with 0.25 Å radius indicating zero confidence and 2.0 Å indicating a confidence of 1.0 [22]. The blue and green toward the right of the protein upon binding of 23–446 peptide shows protection to exchange suggestive of binding in that region. Blue and green closer to the center of the structure in the 23–437 peptide suggests binding in that zone.

HDX revealed major structural changes in the p19 subunit and no interactions in the p40 subunit upon peptide binding. Fig 6 shows the differential HDX analysis of IL-23 in the presence and absence of blocking peptides 23–437 and 23–446. A region in the cytokine, spanning residues 81–90, 89–109 and 143–153 located within the p19 subunit (N-terminal end of Helix B, B-C loop and Helix D) showed significant decreases in deuterium uptake when bound to 23–446. Binding of peptide 23–446 also induced protection from deuterium uptake in residues 15–21 of Helix A. This may be due to a direct binding event or due to an effect upon the kink observed by crystallography (S2 Fig in S1 File) [19] in this helix. Helix A might straighten out when the ligand binds to the other side of p19 subunit, resulting in an increased number and/or strength of hydrogen bonds and therefore increased protection to exchange. Enhanced exchange rate or deprotection from deuterium uptake was also seen in residues 110–115 of Helix C, indicating that part of this helix might have a tendency to unwind. Overall, observed HDX profiles suggest that Helix B, B-C loop and Helix D are the binding interface of peptide 23–446. Protection to exchange observed for peptide 23–437 is on the same residues as for peptides 23–441 and 23–446, and includes helices B, C, and D (S4 Fig in S1 File). Surprisingly, the residues 110–115 that are deprotected by peptide 23–446 are protected by peptide 23–437, suggesting a different binding mode between the two peptides. Although the magnitude of protection differs among the blocking peptides, HDX data confirms the binding region of the blocking peptides to include W137, L141, and K145 (residues marked by arrow. S4 Fig in S1 File), which are the residues determined by phage-yeast binning.

### Depleting out the background binders during selection

The overabundance of FGL-containing peptides in the selected pool made isolation of IL-23 blocking peptides a challenging task. Therefore, we asked if we could use alanine variants of a target displayed on yeast to deplete the libraries from peptides that bind to non-functional sites. In particular, we sorted input round four phage pool against wild-type or ala26 variant of p19 subunit displayed on yeast. Sequence analysis of 96-random phage peptides from the sorted phage pool indicated that FGL-clones were further enriched when wild type IL-23 was used as the target. In contrast, the frequency of FGL-containing peptides was decreased when the same input phage was sorted against Ala26 variant. Comparing frequency of FGL-containing peptides in the two selection arms (wild type IL-23 versus Ala26) suggested a substantial reduction in the number of FGL-containing peptides only if ala26 variant was used as the target (S4 Table in S1 File). Obviously, incorporation of FACS against ala26 at earlier rounds of selection would have been more effective in subtracting FGL-containing peptides since it would have prevented their over-amplification. However, we had not saved any material from earlier rounds of selection. Therefore, we were limited in using input round four. It is also recommended to do multiple rounds of FACS against ala26 without amplifying the phage in between selection rounds to deplete majority of FGL clones. As a result, screening of peptides to identify functional clones that interact with desired regions on the target will became more robust and efficient.

## Discussion

Phage and yeast display platforms are powerful *in vitro* selection techniques for display and isolation of target specific peptides. In this study, we integrated the two platforms by using yeast to display the target of interest and phage to display random peptide libraries. We utilized this phage-against-yeast platform in two ways. First, we screened target specific binders on-phage against alanine variants of target on yeast. This enabled accurate binning of peptides as displayed on-phage based on binding region without the need for peptide chemical synthesis. Second, we streamlined the phage biopanning process by introducing a FACS step to allow efficient discovery of rare hits.

The phage-against-yeast platform was used to study the interaction of IL-23 with IL-23 binding peptides. IL-23 is a heterodimeric cytokine comprised of p19 and p40 subunits and plays a key role in establishing autoimmune diseases such as psoriasis, arthritis, inflammatory bowel disease, and colitis [9–13]. Wild-type IL-23 and alanine variants of the p19 subunit were displayed on yeast. Binding to IL-23 receptor was used as a benchmark to confirm correct assembly of p19 and p40 heterodimer on yeast cells. To identify their binding region(s), peptides on-phage were screened against alanine variants of p19 on yeast using flow cytometry. As a result, only peptides that interact with functional binding region(s) would be synthesized for further characterization. This approach is not only straightforward and accurate; it is also extremely rapid, cost, and resource effective since it eliminated the need for soluble protein [8, 23–25] and chemical peptide synthesis. Importantly, this method can be extended to any protein-protein interaction targets including ligand-receptor pairs. Obviously, mutation of cysteine, glycine, proline, and glycosylation sites on the target [26] should be avoided to prevent conformational change. Although there is a risk of losing allosteric inhibitors, this approach allows sampling the majority of binders and characterizing them based on binding region(s) as displayed on-phage. Therefore, only a limited number of peptides would need synthesis and characterization. In this study, less than twenty peptides (out of 212) were identified as blockers on-phage, five were chemically synthesized, and four inhibited interaction of IL-23 with its receptor as free peptides. Previous work included display of wild-type and alanine variants of antigen/target displayed on yeast and screening them against recombinant antibodies [8, 23, 26–29] or antibodies displayed on phage [24, 25]. To our knowledge, this is the first report describing integration of two different display platforms to map peptides as displayed on-phage based on binding regions using flow cytometry.

We utilized the phage-against-yeast platform to develop and incorporate a FACS step during selection. Traditional methods of biopanning are based on solid and solution based selection. They rely on washing stringencies to discriminate among binders based on affinity [5, 30–32]. Although effective, these methods do not discriminate based on binding region(s). Often during selection, certain phage populations that bind to nonfunctional sites on the target dominate the pool [30], making isolation of rare functional clones a formidable task. To circumvent this challenge, we developed a highly sensitive flow-based phage selection procedure to enrich for clones with defined binding regions. We utilized FACS in later round of selection and were able to reduce the number of background binders. This suggests that incorporation of FACS at earlier rounds would result in a more significant reduction of unwanted clones from the library.

Alternatively, this approach can be used to select for phage clones that interact with a particular binding region. For example, peptides that bind to wild-type IL-23 and not the Ala137 mutation can be identified in an efficient way using FACS from billions of variants. These peptides most possibly would interact with IL-23 where it binds to IL-23R and should be neutralizing. This approach allows sampling of every single variant in the library compared to the commonly practiced phage ELISAs that at best covers 0.1% of the selected pool. In addition, sorting by flow cytometry enables the operator to have control over biopanning parameters and allows real-time selection for affinity, binding region, and function [33–35]. We believe that the strategy described here will greatly enhance the impact of phage display for discovery and development of peptide drug leads.

## Supporting information

S1 File (DOCX)
