# Supplementary material for: Integration of phage and yeast display platforms: A reliable and cost effective approach for binning of peptides as displayed on-phage
Source: PLoS One. 2020 Jun 1;15(6):e0233961. doi: 10.1371/journal.pone.0233961 (PMC7263589; doi:10.1371/journal.pone.0233961)
Supplement: S1 File — (DOCX) [file pone.0233961.s001.docx]

**SUPPORTING INFORMATION**

**MATERIALS & METHODS**

**Crystallization and structure determination**. IL-23 protein for crystallization was generated as previously described [12]. A IL-23 and peptide co-crystal was grown at 8°C using the sitting-drop vapor diffusion method with crystallization drops consisting of 0.3 µL protein (∼16 mg/mL IL-23:peptide mixture with 1:1 molar ratio) and 0.3 µl reservoir solution (100 mM Hepes pH 7, 17% w/v PEG 3350 and 150 mM Potassium Nitrate). The condition generated one crystal after one week. The 100 µm x 100 µm x 50 µm crystal was harvested from an INTELLI-PLATE® 96 well crystallization plate (Art Robbins Instruments) and cryoprotected using well solution supplemented with 20% v/v glycerol and flash-frozen in liquid nitrogen. Diffraction data were collected at LRL-CAT 31-ID (Advanced Photon Source, Argonne, IL). The structure was solved using p19 and p40 subunits from a published structure (Protein Data Bank ID 3DUH) as molecular replacement models in Phaser [13]. Structure refinement followed with iterative rounds of restrained refinement and model building with Buster [14] and COOT [15]. Difference density corresponding to the peptide was observed at the p19:p40 interface and ordered peptide residues were subsequently modeled by hand. The IL-23 structure with peptide was determined to a final resolution of 2.7 Å and belonged to the space group P2_1_2_1_2_1_ with unit cell dimensions of a=73.6 b=94.7 c=101.1 α=β=γ=90. The final refinement R-factors were R_work_=0.206 (0.220) and R_free_=0.241 (0.278) with highest resolution shell in parenthesis (Supplemental Table 1).

**RESULTS**

**IL-23 dynamic behavior in solution:** Digestion of undeuterated IL-23 with pepsin produced excellent sequence coverage for p19 and p40 subunits of IL-23. 56 peptides equating to 97.2% sequence coverage with 4.09 redundancy and 103 peptides equating to 95.8% sequence coverage with 4.17 redundancy were monitored for p19 and p40 subunits, respectively. To form a basis for comparison, the deuterium incorporation rates for IL-23 alone was determined. The p19 D helix, [1] (S1 and 5 Fig) which is in the interface between the two subunits has very little exchange in the p40 binding site. The C-terminal region of helix D away from the binding interface, however, is highly solvent exposed and readily incorporated deuterium. Similarly, the p19 A, B, and C helices show very little D_2_O incorporation in the center of the helix bundle and the unstructured loops. This is especially evident in the C-terminus of the B helix with decreased deuterium uptake. Helices A and C show less protection in the helix core and overall more solvent exposure, signifying a more open structure. D2 and D3 domains in p40 subunit interact with p19 subunit and the regions involved in the interface show relatively little deuterium uptake. The regions of D2 and D3 domains away from the interface appear to be more dynamic and take up more deuterium in a time-based manner. D1 domain in p40 appears to be the most solvent exposed and is more dynamic than the other domains with higher amounts of deuterium incorporation.

**Crystal Structure of Peptide with IL-23:** The crystal structure of IL-23 in complex with 23-652 (PDB ID 6UIB) was determined to confirm the peptide binding location and elucidate specific interactions. Multiple commercially available crystal screens were setup, however, only a single crystal was obtained.  This sole crystal was used to obtain a 2.7 Å structure (S2A Fig and S5 Table). Multiple flexible loops were omitted and several side chains were truncated from the final IL23 model due to lack of sufficient density. Two N-Acetylglucosamine sugars were modeled at Asn200 of p40 with additional positive Fo-Fc density suggesting more sugars were present even though attempts were made to limit glycosylation. Electron density for the peptide was clear enough to model residues 4-18 (missing 3 residues on N-terminus and 2 residues on C-terminus) with the sidechain of Lys5 left unmodeled due to lack of density. 23-652 binds in a V-shaped groove present at the p19 interface with D3 of p40 (S2B Fig). The peptide adopts a di-helical conformation held together by a disulfide bond between Cys8 and Cys14. The first three ordered residues lack secondary structure and are followed by a single turn 3_10_ helix, a short turn, and a two turn α-helix (S2C Fig). The binding region was mapped using MOE [2] and PyMol [3] to reveal 12 residue pairs involved in hydrogen bonding interactions, 1 residue pair involved in a CH-π (arene) interaction and a large number of Van der Waals interactions (S3 Table). All interactions are confined to p19 helix A, a small section of the p19 AB loop and D3 of p40.  Trp26 (p19) flipped out, relative to the apo structures [1, 4], to pick up an interaction with Phe7 of 23-652 (S2D Fig). Arg291 (p19) and Asp270 (p40) also adopted different conformations in order to interact with 23-652 (Gln15) (S2E Fig). The 23-652 binding region is not fully explored in the existing IL23 structures [4-7]. The most relevant IL23 structures include an Adnectin [5] and a Llama nanobody [7] (PDB ID 4GRW). These two molecules overlap with 23-652 N-terminal residues up through Tyr9 (S2F Fig), however they only share one specific interaction (H-bond to p40 Trp297) with 23-652.

**S1 Fig. HDX peptide map of IL-23 in the absence and presence of peptides**. HDX binding map of peptide 23-644 to A) p19 and B) p40 subunits of IL-23. HDX binding map of peptide 23-652 to C) p19 and D) p40 subunits of IL-23. Please note the Y-scale for p40 plots is smaller than the Y-scale for p19.


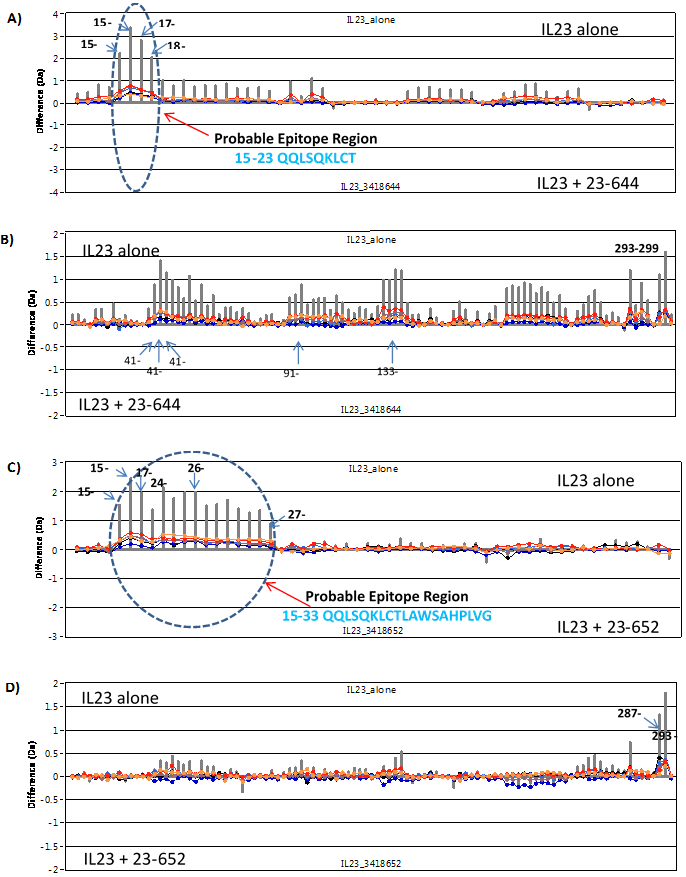


**S2 Fig. IL-23 crystal structure with 23-652 bound.** A) Peptide 23-652 in gold bound to IL-23 P19 subunit in green and P40 subunit in blue. B) 23-652 binds in the V-shaped groove that is formed by the interactions of P19 and P40 D3. C) Ordered residues of 23-652 peptide from complex structure with IL-23. Residues 1-3 and 19-20 were disordered and the sidechain of Lys5 had insufficient density to model. D) IL-23 residues adopt new conformations upon 23-652 binding. Trp26 of p19 in green forms a hydrogen bond with Phe7 backbone of 23-652 in orange. Trp26 of Apo IL-23 (3DUH) in yellow points in towards P19:P40 interface. E) Arg291 and Asp270 of P40 in blue adopt new conformations compared to Apo IL-23 (3DUH in yellow) to interact with Gln15 of 23-652 in orange. F) Adnectin (3QWR) in dark blue and Llama nanobody (4GRW) overlap with the first 9 residues of 23-652. No known structures reach as far into the V-shaped groove of the P19:P40 interface as the α-helix of 23-652.


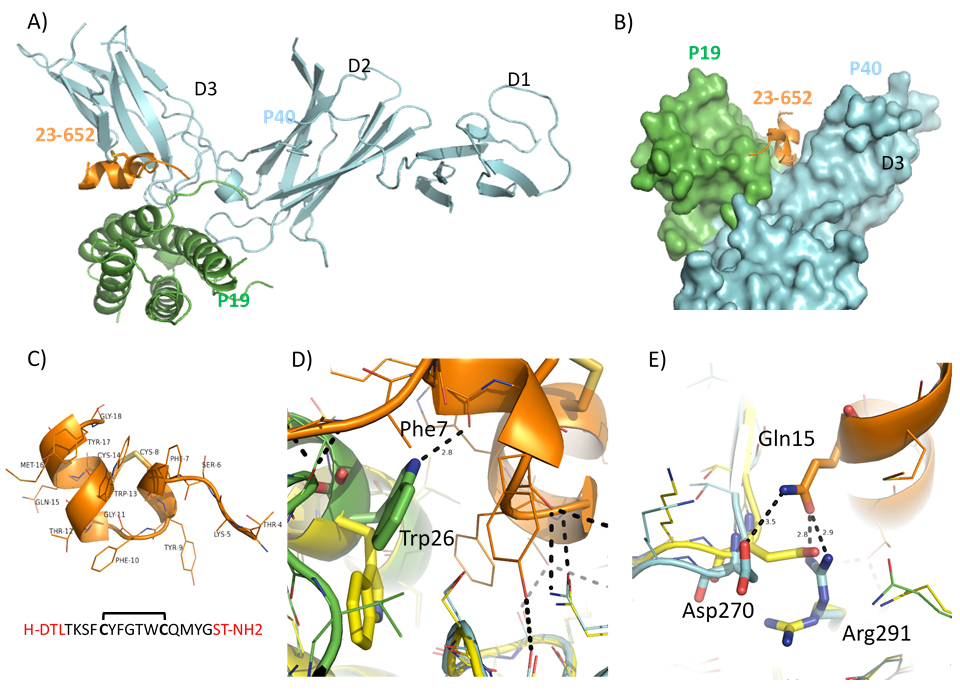


F)


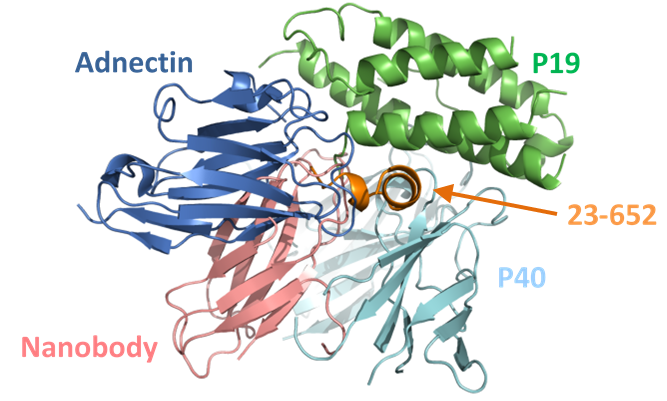


**S3 Fig.** SPR sensorgrams and their cruve fitting for (A) 23-437, (B) 23-441, (C) 23-446, (D) 23-447, (E) 23-644, (F) 23-652, (G) IL23R, and (H) IL23. The experimental sensorgrams were in colors and their fit to a 1:1 binding model were in black lines. The unit of x-axis was time in second; y-axis, response unit (RU).

A B

C D

E F

G H

**S4 Fig.** Sequence of IL23 with color-coded HDX information from the Bayesian analysis. The first row in each panel contains a number to enable easy calculation of residue positions from the remaining rows. Top panel: the Apo state heat map. Log10 rate constants are shown as the background color for each residue; the color scale is indicated at the lower right of this panel. Grey background indicates prolines and other residues for which there was no coverage in the HDX experiment. The remaining panels show the difference between the calculated log10 rate constants for the ligand-bound state and the Apo state for the ligands indicated in the title of each panel. The change in log10 rate constant scale is shown to the lower right of the second panel and is the same for all three lower panels. Helix A spans residues 1-28, Helix B 49-91, Helix C 100-122 and Helix D 137-157; the A-B loop spans 29-47, the B-C loop spans 92-99 and the C-D loop spans 123-137.

**S1 Table: Proteins used in SPR and phage selection.**

| Protein name | Reagent purpose | Description |
| --- | --- | --- |
| IL23-AVI-HIS | IL23 sensor | p19 with C-terminal biotin-labeled AVI and HIS tags complexed with p40 by co-expression in HEK293 cells |
| IL23-HIS | Analyte | p19 with HIS tag complexed with p40 by co-expression in HEK293 cells |
| IL23R-Fc-AVI-HIS | IL23R sensor | IL23R ECD (aa 1-353) fused to Fc followed by biotin-labeled AVI and HIS tags produced in HEK293 cells |
| IL23R-FLAG-HIS | Analyte | IL23R ECD (aa 1-353) with C-terminal Flag and HIS tags produced in HEK293 cells |

**S2 Table: Library Specification**

| **Library Specification** | | |
| --- | --- | --- |
| Library |  | Diversity |
| 282 | X_2_CX_8_CX_2_ | 1 X 10^8^ |
| 555 | X_5_CX_5_CX_5_ | 6 X 10^9^ |
| 666 | X_6_CX_6_CX_6_ | 7.5 X 10^9^ |
| 15-mer Linear | X_15_ | 4.6 X 10^9^ |

**S3 Table: Specific interactions generated by MOE and PyMol**

| Type | Peptide | Peptide Residue | IL-23 | IL-23 Residue | Distance (Angstroms) | Interaction Pairs^$^ |
| --- | --- | --- | --- | --- | --- | --- |
| hbond | 23-652 | Thr4 | P19 | Pro30 | 3.3 | s-b |
| hbond | 23-652 | Lys5 | P19 | Pro30 | 2.9 | b-b |
| hbond | 23-652 | Phe7 | P19 | Trp26 | 2.8 | b-s |
| hbond | 23-652 | Tyr9 | P40 | Gln289 | 3.3 | b-s |
| hbond | 23-652 | Tyr9 | P40 | Ser294 | 3.3 | s-s |
| hbond | 23-652 | Tyr9 | P40 | Trp297 | 2.7 | b-s |
| hbond | 23-652 | Phe10 | P40 | Gln289 | 3.5 | b-s |
| hbond | 23-652 | Gly11 | P40 | Gln289 | 2.8 | b-s |
| hbond | 23-652 | Thr12 | P40 | Thr250 | 2.9 | s-s |
| hbond | 23-652 | Thr12 | P40 | Arg291 | 2.7 | s-b |
| arene | 23-652 | Cys14 | P40 | Phe268 | 3.7 | s-s |
| hbond | 23-652 | Gln15 | P40 | Asp270 | 3.5 | s-s |
| hbond | 23-652 | Gln15 | P40 | Arg291 | 2.8 | s-s |
| hbond | 23-652 | Gln15 | P40 | Arg291 | 2.9 | s-s |

^$^ s: side chain and b: backbone

**S4 Table: %FGL containing peptides prior and after sort against Ala26.** Input round five phage was selected against wild type IL-23 and Ala26 variant of p19 displayed on yeast. The percentage of the FGL-containing prior and after sort is shown below. Population of FGL containing peptides were increased if the library was sorted against wild type IL-23. In contrast, FGL containing peptides were significantly depleted out of library when the sort was conducted against Ala26 variant on yeast.

| **Library** | **%FGL containing peptides**  **Input rd5** | **%FGL containing peptides**  **Output rd5** | |
| --- | --- | --- | --- |
|  |  | Sorted against wt-IL-23 on yeast | Sorted against Ala26 on yeast |
| 555 | 66 | 73 | 58 |
| 666 | 59 | 88 | 55 |

**S5 Table: Data collection and refinement statistics**

| Data collection | LRL-CAT (APS 31-ID) |
| --- | --- |
| Space group | P2_1_2_1_2_1_ |
| Unit cell a, b, c (Å) | 73.6, 94.7, 101.1 |
| α, β, γ (°) | 90, 90, 90 |
| Resolution range (Å) | 101.1-2.74 |
| Measured reflections | 135345 |
| Unique reflections | 18723 |
| Completeness (%) | 97.6 (100) |
| Multiplicity | 7.2 (7.5) |
| Mean I/σ(I) | 23.9 (4.3) |
| Rsym | 0.050 (0.428) |
| **Rrefinement statistics** |  |
| Refinement resolution (Å) | 19.90-2.74 |
| Reflections used | 18625 |
| Completeness (%) | 97.5 (100) |
| R (%) | 20.6 (22.0) |
| R_free_ (%) | 24.1 (27.8) |
| Mean B-factor (Å^2^) | 84.89 |
| RMSD bonds (Å) | 0.009 |
| RMSD angles (°) | 1.15 |
| The highest resolution shell is shown in parentheses | |

**References:**

1. Lupardus PJ, Garcia, K.C. The structure of interleukin-23 reveals the molecular basis of p40 subunit sharing with interleukin-12. Journal of Molecular Biology. 2008;382:931-41. doi: DOI:10.1016/j.jmb.2008.07.

2. Chemical Computing Group ULC SSW, Suite #910, Montreal, QC, Canada, H3A 2R7. Molecular Operating Environment (MOE). 2017;2013.08.

3. The PyMOL Molecular Graphics System VS, LLC. PyMol.

4. Beyer BM, Ingram, R., Ramanathan, L., Reichert, P., Le, H.V., Madison, V., Orth, P. Crystal structures of the pro-inflammatory cytokine interleukin-23 and its complex with a high-affinity neutralizing antibody. Journal of Molecular Biology. 2008;382:942-55. doi: PubMed ID 18708069 DOI:10.1016/j.jmb.2008.08.

5. Ramamurthy V, Krystek, S.R., Bush, A., Wei, A., Emanuel, S.L., Das Gupta, R., Janjua, A., Cheng, L., Murdock, M., Abramczyk, B., Cohen, D., Lin, Z., Morin, P., Davis, J.H., Dabritz, M., McLaughlin, D.C., Russo, K.A., Chao, G., Wright, M.C., Jenny, V.A., Engle, L.J., Furfine, E., Sheriff, S. Structures of adnectin/protein complexes reveal an expanded binding footprint. Structure. 2012;20:259-69. doi: DOI:10.1016/j.str.2011.11.0.

6. Desmet J, Verstraete, K., Bloch, Y., Lorent, E., Wen, Y., Devreese, B., Vandenbroucke, K., Loverix, S., Hettmann, T., Deroo, S., Somers, K., Henderikx, P., Lasters, I., Savvides, S.N. Structural Basis Of Il-23 Antagonism By An Alphabody Protein Scaffold. Nat Commun. 2014;5:5237.

7. Desmyter A, Spinelli, S., Button, C., Saunders, M., de Haard, H., Rommelaere, H., Union, A., Cambillau, . Potent Multitopic Nanobody Inhibitors of the Chronic Inflammation Promoting Cytokine hIL23. doi: DOI: 10.2210/pdb4grw/pdb.
